# Inhibition of G Protein-Activated Inwardly Rectifying K^+^ Channels by Different Classes of Antidepressants

**DOI:** 10.1371/journal.pone.0028208

**Published:** 2011-12-02

**Authors:** Toru Kobayashi, Kazuo Washiyama, Kazutaka Ikeda

**Affiliations:** 1 Department of Project Programs, Center for Bioresource-based Researches, Brain Research Institute, Niigata University, Niigata, Japan; 2 Research Project for Addictive Substances, Tokyo Metropolitan Institute of Medical Science, Tokyo, Japan; Chiba University Center for Forensic Mental Health, Japan

## Abstract

Various antidepressants are commonly used for the treatment of depression and several other neuropsychiatric disorders. In addition to their primary effects on serotonergic or noradrenergic neurotransmitter systems, antidepressants have been shown to interact with several receptors and ion channels. However, the molecular mechanisms that underlie the effects of antidepressants have not yet been sufficiently clarified. G protein-activated inwardly rectifying K^+^ (GIRK, Kir3) channels play an important role in regulating neuronal excitability and heart rate, and GIRK channel modulation has been suggested to have therapeutic potential for several neuropsychiatric disorders and cardiac arrhythmias. In the present study, we investigated the effects of various classes of antidepressants on GIRK channels using the *Xenopus* oocyte expression assay. In oocytes injected with mRNA for GIRK1/GIRK2 or GIRK1/GIRK4 subunits, extracellular application of sertraline, duloxetine, and amoxapine effectively reduced GIRK currents, whereas nefazodone, venlafaxine, mianserin, and mirtazapine weakly inhibited GIRK currents even at toxic levels. The inhibitory effects were concentration-dependent, with various degrees of potency and effectiveness. Furthermore, the effects of sertraline were voltage-independent and time-independent during each voltage pulse, whereas the effects of duloxetine were voltage-dependent with weaker inhibition with negative membrane potentials and time-dependent with a gradual decrease in each voltage pulse. However, Kir2.1 channels were insensitive to all of the drugs. Moreover, the GIRK currents induced by ethanol were inhibited by sertraline but not by intracellularly applied sertraline. The present results suggest that GIRK channel inhibition may reveal a novel characteristic of the commonly used antidepressants, particularly sertraline, and contributes to some of the therapeutic effects and adverse effects.

## Introduction

Depression is one of the most common illnesses in the world [1], [2]. After the efficacy of tricyclic antidepressants (TCAs), including imipramine, amitriptyline and amoxapine, was well established, various classes of antidepressants were introduced, including selective serotonin reuptake inhibitors (SSRIs; fluoxetine, paroxetine and sertraline), serotonin-norepinephrine reuptake inhibitors (SNRIs; venlafaxine and duloxetine), selective norepinephrine reuptake inhibitors (NRIs; reboxetine), noradrenergic and specific serotonergic antidepressants (NaSSAs; mirtazapine and mianserin), and 5-hydroxytryptamine type 2 (5-HT_2_) receptor antagonists (nefazodone) [1]–[3]. Antidepressants are commonly used for the treatment of depression and several neuropsychiatric disorders, such as anxiety disorders, eating disorders, obsessive-compulsive disorders, and chronic pain disorders [1]–[3]. Their clinical efficacy is hypothesized to be linked mainly with facilitation of noradrenergic or serotonergic function in the brain [2]. In contrast, the interaction between antidepressants and muscarinic, α_1_ adrenergic, and H_1_ histamine receptors is involved in some of their adverse side effects, such as dry mouse, orthostatic hypotension, and sedation [2]. Antidepressants have also been shown to modulate the function of several other receptors and ion channels, including 5-HT_2C_ and 5-HT_3_ receptors, nicotinic acetylcholine receptors, *N*-methyl-d-aspartate (NMDA) receptor channels, P2X_2_ receptors, voltage-gated Ca^2+^, Na^+^, and K^+^ channels, Ca^2+^-activated K^+^ channels, two-pore-domain K^+^ channels, and volume regulated anion channels [4]–[23]. The modulation of these receptors and channels might also be relevant to the pharmacological effects of antidepressants. However, the molecular mechanisms that underlie the effects of various antidepressants have not yet been sufficiently clarified.

G protein-activated inwardly rectifying K^+^ (GIRK) channels (also known as Kir3 channels) are members of a major subfamily of inwardly rectifying K^+^ (Kir) channels that includes seven subfamilies [24]. Four GIRK channel subunits have been identified in mammals [25]–[27]. Neuronal GIRK channels are predominantly heterotetramers composed of GIRK1 and GIRK2 subunits in most brain regions or homotetramers composed of GIRK2 subunits in the substantia nigra [27]–[30], whereas atrial GIRK channels are heterotetramers composed of GIRK1 and GIRK4 subunits [26]. The channels are activated by various G_i/o_-protein-coupled receptors, such as M_2_ muscarinic, α_2_ adrenergic, D_2_ dopaminergic, opioid, nociceptin/orphanin FQ, CB_1_ cannabinoid, and A_1_ adenosine receptors, through the direct action of G-protein βγ subunits [31]–[33]. Additionally, ethanol activates GIRK channels independently of G-protein-coupled signaling pathways [34], [35]. GIRK channels play an important role in regulating neuronal excitability, synaptic transmission, and heart rate [31], [36]–[39]. Furthermore, recent studies have suggested that GIRK channel modulation has the potential for treating several neuropsychiatric disorders and cardiac arrhythmias [33], [40], [41]. Therefore, GIRK channel modulators may affect various brain and cardiac functions. We have demonstrated the distinctive effects of several antidepressants on GIRK channels, even among the same class, particularly SSRIs [42], [43]. To further clarify the interaction between various classes of commonly used antidepressants and GIRK channels may be useful for advancing our understanding of the pharmacological effects of antidepressants. In the present study, we examined the effects of various antidepressants on GIRK channels using the *Xenopus* oocyte expression assay.

## Materials and Methods

### Preparation of specific mRNAs

Plasmids that contain the entire coding sequences for the mouse GIRK1, GIRK2, and GIRK4 channel subunits were obtained previously [34], [44], [45]. cDNAs for mouse Kir2.1 in pcDNA1 [46] were generously provided by Dr. Lily Y. Jan (University of California, San Francisco). These plasmids were linearized by digestion with the appropriate enzymes as described previously [45], [46]. The specific mRNAs were synthesized *in vitro* using the mMESSAGE mMACHINE™ *In Vitro* Transcription Kit (Ambion, Austin, TX, USA).

### Electrophysiological analysis

Adult female *Xenopus laevis* frogs (Copacetic, Soma, Aomori, Japan) were anesthetized by immersion in water that contained 0.15% tricaine (Sigma-Aldrich, St. Louis, MO, USA). A small incision was made on the abdomen to remove several ovarian lobes from the frogs, which were humanely killed after the final collection. All procedures for the care and treatment of animals were performed in accordance with National Institutes of Health guidelines and were approved by the Institutional Animal Care and Use Committee of Niigata University (Permit Number: 172-2). *Xenopus* oocytes (Stages V and VI) were manually isolated from the ovary and maintained in Barth's solution [47]. Oocytes were injected with mRNA for GIRK1/GIRK2 or GIRK1/GIRK4 combinations (0.15 ng each) or Kir2.1 (0.3 ng). The oocytes were incubated at 19°C in Barth's solution and manually defolliculated after treatment with 0.8 mg/ml collagenase (Wako Pure Chemical Industries, Osaka, Japan) for 1 h. The whole-cell currents of the oocytes were recorded from 3 to 9 days after injection with a conventional two-electrode voltage clamp [34], [48]. The membrane potential was held at −70 mV unless otherwise specified. Microelectrodes were filled with 3 M KCl. The oocytes were placed in a 0.05 ml narrow chamber and continuously superfused with a high-potassium (hK) solution (96 mM KCl, 2 mM NaCl, 1 mM MgCl_2_, 1.5 mM CaCl_2_ and 5 mM HEPES, pH 7.4 with KOH) or a K^+^-free high-sodium (ND98) solution (98 mM NaCl, 1 mM MgCl_2_, 1.5 mM CaCl_2_ and 5 mM HEPES, pH 7.4 with NaOH) at a flow rate of 2.5 ml/min. In the hK solution, the K^+^ equilibrium potential was close to 0 mV, and the inward K^+^ current flow through the Kir channels was observed at negative holding potentials as previously shown [25], [27], [43]. Additionally, to examine the effects of antidepressants on outward K^+^ currents, a perfusion solution that contained 4 mM K^+^ (K4 solution) was made by substituting NaCl with KCl in the ND98 solution. To examine the effects of an antidepressant on GIRK channels activated by G-protein activation, 13.8 nl of 100 mM Li_4_-guanosine-5′-*O*-(3-thiotriphosphate) (GTPγS; Sigma-Aldrich), a nonhydrolyzable G-protein activator, dissolved in distilled water was injected into an oocyte using a nanoliter injector (World Precision Instruments, Sarasota, FL, USA) as described previously [49]. Furthermore, to examine the effects of intracellular sertraline, 23 nl of 10 mM sertraline dissolved in distilled water was injected into an oocyte using a Nanoliter injector as described previously [50], and the oocyte currents were then continuously recorded for approximately 30–40 min. Because the volume of the *Xenopus* oocytes used was approximately 1 µl, the intracellular concentration of sertraline was presumed to be approximately 225 µM. For the analysis of concentration-response relationships, the data were fitted to a standard logistic equation [51] using KaleidaGraph (Synergy Software, Reading, PA, USA). The concentration of a drug that produces 50% of the maximal current response for that drug (EC_50_), the concentrations required to reduce control currents by 25% and 50% (IC_25_ and IC_50_, respectively), and the Hill coefficient (*n*_H_) were obtained from the concentration-response relationships.

### Data analyses

The data are expressed as mean ± SEM, and *n* is the number of oocytes tested. The statistical analysis of differences between groups was performed using paired *t*-test, one-way analysis of variance (ANOVA), or two-way ANOVA followed by the Tukey-Kramer *post hoc* test. Values of *P*<0.05 were considered statistically significant.

### Compounds

All of the antidepressants tested were commercially purchased. Amoxapine and nefazodone hydrochloride were obtained from Sigma-Aldrich. Mirtazapine and mianserin hydrochloride were obtained from Tocris Bioscience (Bristol, UK). Sertraline hydrochloride and duloxetine hydrochloride were obtained from Tronto Research Chemicals (North York, Canada). Venlafaxine hydrochloride was obtained from LKT Laboratories (St. Paul, MN, USA). Sertraline was dissolved in dimethyl sulfoxide (DMSO) or distilled water, and venlafaxine was dissolved in distilled water. The other antidepressants were dissolved in DMSO. The stock solution of each compound was stored at −30°C until use. Ethanol was purchased from Wako Pure Chemical Industries. Each compound was added to the perfusion solution in appropriate amounts immediately before the experiments.

## Results

### Inhibition of GIRK channels by antidepressants

**Figure 1:**
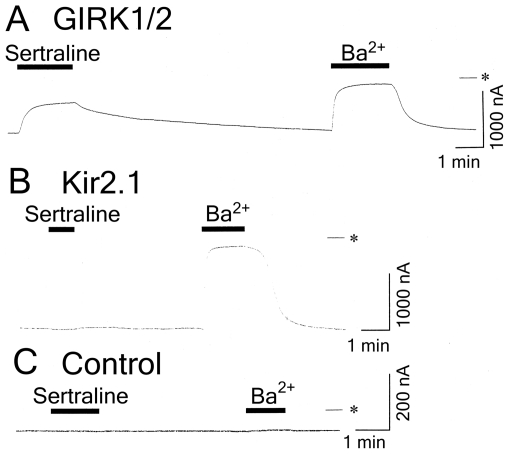
Inhibitory effects of sertraline on GIRK channels expressed in *Xenopus* oocytes. (A) In an oocyte injected with GIRK1 and GIRK2 mRNAs, current responses to 30 µM sertraline and 3 mM Ba^2+^ are shown. (B) In an oocyte injected with Kir2.1 mRNA, current responses to 100 µM sertraline and 3 mM Ba^2+^ are shown. (C) In an uninjected oocyte, no significant current responses to 300 µM sertraline or 3 mM Ba^2+^ are shown. Current responses were measured at a membrane potential of −70 mV in an hK solution that contained 96 mM K^+^. Asterisks show the zero current level. Horizontal bars indicate the duration of application.

In *Xenopus* oocytes injected with GIRK1 and GIRK2 mRNAs, basal GIRK currents, which depend on free G-protein βγ subunits present in the oocytes because of the inherent activity of G-proteins [32], were observed at a holding potential of −70 mV in an hK solution that contained 96 mM K^+^ (Fig. 1A). The 3 mM Ba^2+^-sensitive current components (1042.8±90.1 nA, *n* = 30) correspond to the magnitude of GIRK currents in oocytes that express GIRK channels [34]. Extracellular application of 30 µM sertraline, an SSRI, reversibly reduced the inward currents through the expressed GIRK channels (Fig. 1A). The current responses to an additional 100 µM sertraline during the application of 3 mM Ba^2+^, which blocks Kir channels, were not significant (reduction of inward currents by 4.5±3.5 nA; less than 1% inhibition of the Ba^2+^-sensitive current components, *n* = 4). Sertraline at 100 µM produced no significant response in a K^+^-free ND98 perfusion solution that contained 98 mM Na^+^ instead of the hK solution (3.0±1.8 nA, *n* = 4), suggesting that the SSRI-sensitive current components show K^+^ selectivity. Additionally, the application of DMSO or distilled water, the solvent vehicles, at the highest concentration (0.3%) induced no significant current response in the hK or ND98 solutions (*n* = 5; data not shown). In contrast, in oocytes injected with mRNA for Kir2.1, a constitutively active Kir channel [46], extracellular application of 300 µM sertraline had no significant effect on the inward currents through the channels in the hK solution (less than 2% change of the Ba^2+^-sensitive current components; 848.3±322.0 nA, *n* = 4; Fig. 1B). In uninjected oocytes, 300 µM sertraline and 3 mM Ba^2+^ caused no significant response (2.0±2.0 nA, *n* = 4, and 3.1±1.7 nA, *n* = 4, respectively; Fig. 1C) compared with oocytes injected with GIRK mRNA, suggesting no significant effect of sertraline or Ba^2+^ on intrinsic oocyte channels. Furthermore, in oocytes injected with GIRK1 and GIRK4 mRNAs, 30 µM sertraline similarly inhibited basal GIRK currents under the same conditions (51.6±4.3% inhibition of 3 mM Ba^2+^-sensitive current components, 561.7±58.2 nA, *n* = 11). Additionally, the Ba^2+^-sensitive current components in oocytes injected with mRNA for GIRK1/GIRK2 or GIRK1/GIRK4 combinations were very significantly larger than those in oocytes injected with the same small amount of a single GIRK mRNA (less than 20 nA, *n* = 7, respectively). The results indicate that sertraline predominantly inhibited GIRK1/2 and GIRK1/4 heteromultimeric channels, but not Kir2.1 channels. Moreover, the effects of different classes of antidepressants on GIRK channels were examined using the same expression assay. Amoxapine, a second generation TCA, and duloxetine, an SNRI, significantly inhibited basal GIRK currents at 100 µM (45.6±4.4 and 65.6±1.0% inhibition for GIRK1/2, *n* = 5 and 7, respectively; 27.6±4.0 and 49.7±2.2% inhibition for GIRK1/4, *n* = 4 and 6, respectively). However, the 5-HT_2_ receptor antagonist nefazodone, NaSSAs mianserin and mirtazapine, and SNRI venlafaxine weakly inhibited the currents at 100 µM (35.9±3.5, 24.1±5.5, 17.6±3.5, and 19.4±4.6% inhibition for GIRK1/2, *n* = 11, 4, 4, and 4, respectively; 30.3±3.4, 18.8±1.3, 12.1±2.4, and 22.8±3.2% inhibition for GIRK1/4, *n* = 13, 5, 5, and 5, respectively). Additionally, the inhibitions were reversible with washout, similar to sertraline (data not shown). In contrast, Kir2.1 channels were insensitive to these drugs at 100 µM (less than 4% change of the Ba^2+^-sensitive current components; 912.5±182.8 nA, *n* = 4). In uninjected oocytes, 300 µM of the drugs caused no significant response (less than 6 nA; *n* = 4 for each of the drugs). Altogether, the results suggest significant inhibition of GIRK channels by sertraline, duloxetine, and amoxapine, weak inhibition of the channels by nefazodone, mianserin, mirtazapine, and venlafaxine, and no significant effects of the drugs on Kir2.1 channels.

### Concentration-dependent inhibition of GIRK channels by various antidepressants

**Figure 2:**
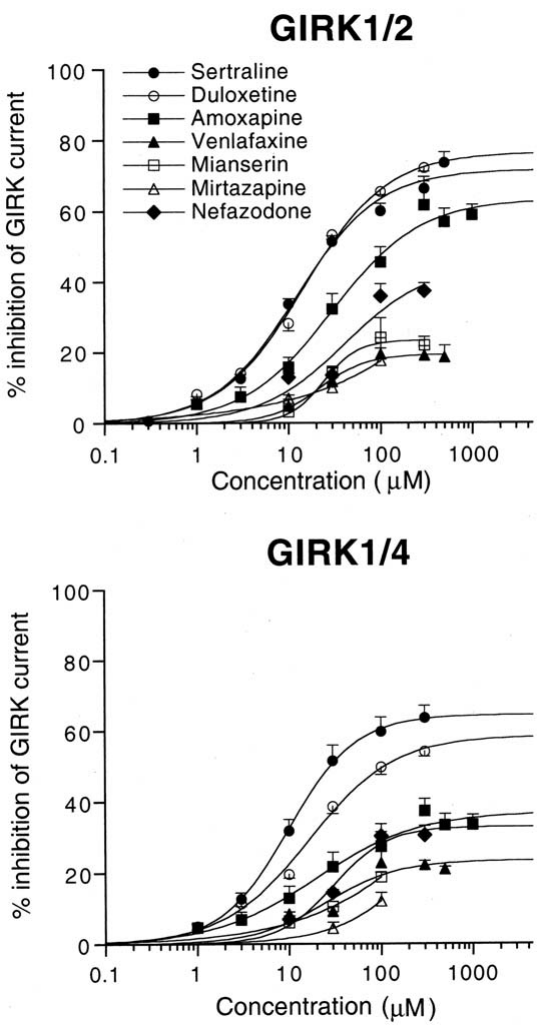
Concentration-response relationships for the effects of various antidepressants on GIRK1/2 and GIRK1/4 channels. The magnitudes of inhibition of GIRK currents by the drugs were compared with the 3 mM Ba^2+^-sensitive current components in oocytes that expressed GIRK1/2 channels or GIRK1/4 channels (762.8±36.0 nA, *n* = 50, and 585.0±44.0 nA, *n* = 40, respectively). Each point and error bar represent the mean ± SEM of the percentage responses.

The concentration-response relationships for the inhibitory effects of different classes of antidepressants on GIRK1/2 and GIRK1/4 channels were investigated. Figure 2 shows that the inhibitions of both types of GIRK channels by various antidepressants were concentration-dependent with distinctive potency and effectiveness at micromolar concentrations. The rank order of the inhibition of GIRK channels by 100 µM of these drugs was the following: duloxetine≥sertraline>amoxapine>nefazodone>mianserin≥venlafaxine≈mirtazapine for GIRK1/2 channels and sertraline>duloxetine≫nefazodone, amoxapine>venlafaxine, mianserin>mirtazapine for GIRK1/4 channels. Table 1 shows the EC_50_ and *n*_H_ values obtained from the concentration-response relationships for sertraline, duloxetine and amoxapine, and the percentage inhibition of the GIRK currents by the drugs at the highest concentrations tested. Additionally, because the drugs could not completely block these types of GIRK channels even at the highest concentrations tested, the IC_25_ and IC_50_ values were also calculated to further compare the effects of the drugs (Table 1). The inhibition of GIRK1/2 channels by sertraline was similar to that by duloxetine (Fig. 2). Furthermore, the inhibition of GIRK1/2 channels by sertraline was statistically similar to the inhibition of GIRK1/4 channels (*P*>0.05 at each concentration, Tukey-Kramer *post hoc* test; Fig. 2, Table 1). In contrast, the inhibition of GIRK1/2 channels by duloxetine and amoxapine was more effective than the inhibition of GIRK1/4 channels (*P*<0.05 at 30, 100, and 300 µM for duloxetine and *P*<0.05 at 300, 500, and 1000 µM for amoxapine, Tukey-Kramer *post hoc* test; Fig. 2, Table 1).

**Table 1:** Inhibitory effects of sertraline, duloxetine and amoxapine on GIRK channels.

	*Sertraline*		*Duloxetine*		*Amoxapine*	
	GIRK1/2	GIRK1/4	GIRK1/2	GIRK1/4	GIRK1/2	GIRK1/4
EC_50_ (µM)	11.7±1.0	12.6±2.5	14.9±0.4	17.0±1.3	38.7±6.2	17.7±4.4
IC_25_ (µM)	6.9±0.6	7.0±1.0	6.6±0.6	12.6±1.2	21.5±8.3	39.7±15.8
IC_50_ (µM)	29.1±3.4	36.7±7.8	28.3±2.5	124.2±34.3	181.1±48.3	ND
% max	73.7±2.9	63.7±3.5	72.2±1.1	54.1±1.5	58.9±2.9	36.0±1.6
(µM; *n*)	(500; 16)	(300; 11)	(300; 7)	(300; 6)	(1000; 5)	(1000; 4)
*n*_H_	1.02±0.05	0.89±0.09	0.94±0.06	0.97±0.07	0.87±0.03	0.87±0.07

Mean ± SEM concentrations of antidepressants (µM) that produce 50% of the maximal effect (EC_50_) and are required to reduce basal GIRK currents by 25% and 50% (IC_25_ and IC_50_, respectively) are shown. The % max values indicate the mean ± SEM percentage inhibition of basal GIRK currents by a drug at the highest concentrations tested. The highest concentrations tested (µM) and the number of oocytes tested (*n*) are shown in parentheses. The *n*_H_ values indicate the mean ± SEM of Hill coefficients. ND indicates that the value was not determined because of a low effectiveness of the drug.

### Characteristics of inhibition of GIRK channels by the SSRI sertraline and SNRI duloxetine

**Figure 3:**
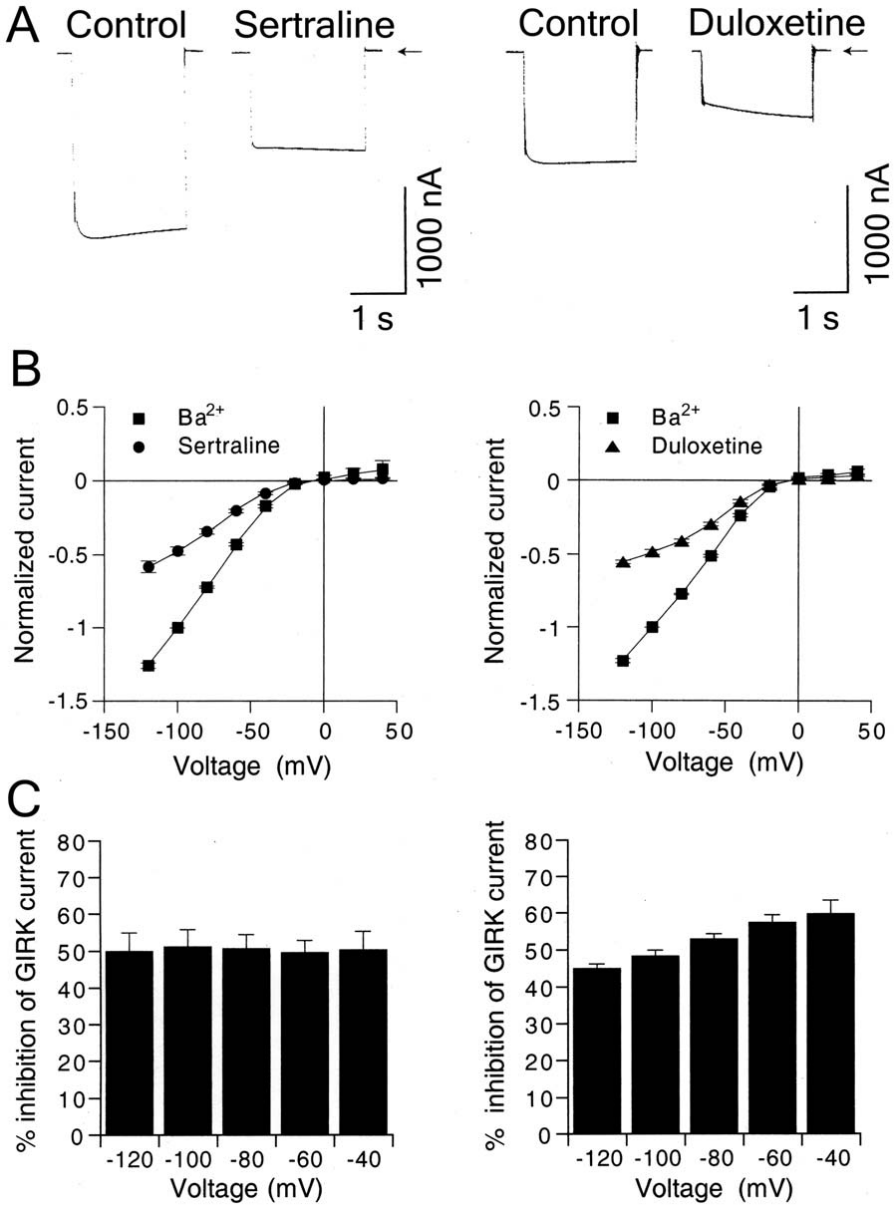
Characteristics of the inhibitory effects of sertraline and duloxetine on GIRK currents. (A) Representative GIRK1/2 currents elicited by a voltage step to −100 mV for 2 s from a holding potential of 0 mV in the presence or absence of 30 µM sertraline (left) or 30 µM duloxetine (right) applied for 3 min. Current responses were recorded in an hK solution that contained 96 mM K^+^. Arrows indicate the zero current level. (B) Current-voltage relationships of the magnitudes of 3 mM Ba^2+^-sensitive currents and the magnitudes of currents reduced by 30 µM sertraline (left, *n* = 9) or 30 µM duloxetine (right, *n* = 6) in oocytes that expressed GIRK1/2 channels. Current responses were normalized to the 3 mM Ba^2+^-sensitive current component measured at a membrane potential of −100 mV (1851.0±220.4 nA, *n* = 15). (C) Percentage inhibition of GIRK1/2 channels by 30 µM sertraline or 30 µM duloxetine over the voltage range of −120 to −40 mV. The magnitudes of inhibition of GIRK currents by 30 µM sertraline (left, *n* = 9) and duloxetine (right, *n* = 6) at the end of the voltage pulses were compared with the 3 mM Ba^2+^-sensitive current components. All values are expressed as mean ± SEM.

Sertraline and duloxetine, which belong to commonly used classes of antidepressants, effectively inhibited GIRK channels, and we further investigated the effects of these drugs in more detail. Instantaneous GIRK1/2 currents elicited by the voltage step to −100 mV from a holding potential of 0 mV were diminished in the presence of 30 µM sertraline applied for 3 min (Fig. 3A). The percentage inhibition of the steady-state GIRK current at the end of the voltage step by sertraline was not significantly different from that of the instantaneous current (*P*>0.05, paired *t*-test; *n* = 9 at −40, −60, −80, −100, and −120 mV, respectively). For duloxetine, the instantaneous currents were primarily diminished in the presence of 30 µM duloxetine, and the currents gradually increased in the voltage step (Fig. 3A). The percentage inhibition of the steady-state GIRK current at the end of the voltage step by duloxetine significantly decreased compared with that of the instantaneous current (*P*<0.05 at −80, −100 and −120 mV, paired *t*-test, *n* = 6). Figure 3B shows that 30 µM sertraline- and duloxetine-sensitive currents in oocytes that expressed GIRK1/2 channels increased with negative membrane potentials, and the current-voltage relationships showed strong inward rectification (*n* = 9 and 6, respectively), similar to 3 mM Ba^2+^-sensitive currents that corresponded to basal GIRK currents, indicating a characteristic of GIRK currents. The percentage inhibition of GIRK1/2 currents by 30 µM sertraline at the end of the voltage pulses showed no significant difference across voltages between −120 and −40 mV (no significant sertraline effect×membrane potential effect interaction, *P*>0.1, one-way ANOVA; *P*>0.1 across voltages, Tukey-Kramer *post hoc* test; Fig. 3C), suggesting voltage-independent inhibition of GIRK channels by sertraline. In contrast, the GIRK current inhibition by duloxetine at the end of the voltage pulses was voltage-dependent, with weaker inhibition at more negative membrane potentials (significant duloxetine effect×membrane potential effect interaction, *P*<0.05, one-way ANOVA; significant differences between −120 and −60 mV, between −120 and −40 mV, between −100 and −60 mV, and between −100 and −40 mV, *P*<0.05, Tukey-Kramer *post hoc* test, *n* = 6, Fig. 3C). The voltage-dependency was associated with a time-dependent decrease in the inhibition by duloxetine in the voltage pulses at more negative membrane potentials. Furthermore, similar results were obtained in oocytes that expressed GIRK1/4 channels (*n* = 4 for each of the drugs; data not shown). Altogether, sertraline and duloxetine primarily inhibited GIRK channels at the holding potential of 0 mV before the voltage pulses. The inhibitory effects of sertraline were voltage-independent and time-independent during each voltage pulse, whereas those of duloxetine decreased voltage-dependently with negative membrane potentials and time-dependently up to a steady state current level in each voltage pulse.

Furthermore, the effects of the two antidepressants on GIRK channels under a physiological K^+^ condition were examined. In oocytes injected with GIRK1 and GIRK2 mRNAs, outward currents observed at a holding potential of −10 mV in a K4 solution that contained 4 mM K^+^ were reversibly reduced by 30 µM sertraline (*n* = 4), 30 µM duloxetine (*n* = 4), and 3 mM Ba^2+^ (the Ba^2+^-sensitive current components, 49.0±2.8 nA, *n* = 8; Fig. S1), whereas in uninjected oocytes, the drugs at 100 µM and 3 mM Ba^2+^ caused no significant response (3.0±0.9 nA for sertraline, 0±0 nA for duloxetine, and 7.6±1.3 nA for Ba^2+^; *n* = 4, 4, and 8, respectively). The results suggest that the antidepressants also inhibited outward GIRK currents at a physiologically extracellular K^+^ concentration.

**Figure 4:**
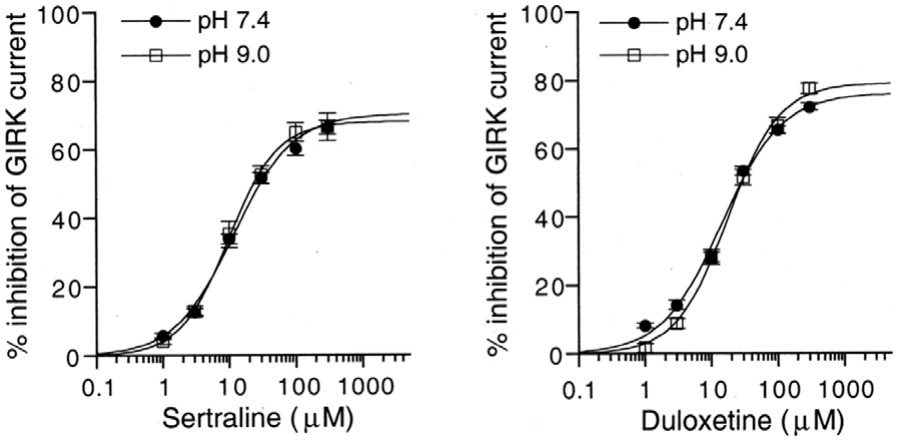
Concentration-dependent inhibition of GIRK channels by sertraline or duloxetine at different pH values. The magnitudes of inhibition of GIRK currents by the antidepressants were compared with the 3 mM Ba^2+^-sensitive current components in oocytes that expressed GIRK1/2 channels (1020.8±96.2 nA at pH 7.4, *n* = 16 for sertraline and *n* = 7 for duloxetine; 1079.5±173.8 nA at pH 9.0, *n* = 7 for sertraline and *n* = 6 for duloxetine, respectively). Current responses were measured at a membrane potential of −70 mV in an hK solution that contained 96 mM K^+^. Each point and error bar represent the mean ± SEM of the percentage responses.

Sertraline and duloxetine possess a secondary amine group with pK_a_ values of 8.9 and 9.34, respectively (Data Sheets of Pfizer and Eli Lilly and Company). At physiological pH or below, sertraline and duloxetine exist mainly in a protonated form, approximately 96.9% and 98.9% at pH 7.4, respectively, and the proportion of the uncharged form increases with an increase in pH. We examined whether changes in extracellular pH would affect GIRK channel inhibition by sertraline or duloxetine. However, in oocytes that expressed GIRK1/2 channels, the percentage inhibition of GIRK channels by sertraline or duloxetine at the same concentrations was not significantly affected by extracellular pH 7.4 and 9.0 (no significant pH×drug interaction, *P*>0.05, two-way ANOVA; *P*>0.05 at each concentration, Tukey-Kramer *post hoc* test; Fig. 4). The results indicate that a marked increase in the proportion of the uncharged form of sertraline and duloxetine may not significantly affect all of the inhibitory effects on GIRK channels, suggesting that GIRK channel inhibition may be mediated by both forms of the drugs with similar effectiveness. Additionally, the inhibition by the antidepressants was unlikely mediated by nonspecific membrane perturbation induced by the uncharged form.

### Effects of sertraline on GIRK channels activated by GTPγS, a nonhydrolyzable GTP

**Figure 5:**
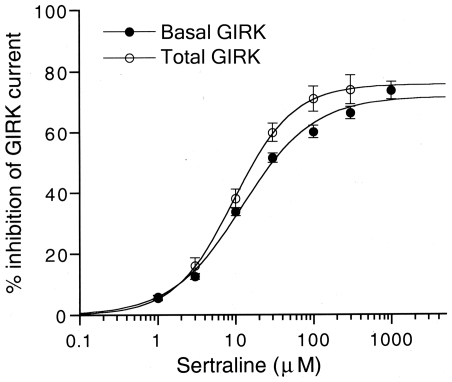
Effects of sertraline on total GIRK currents composed of GTPγS-induced and basal GIRK currents. For comparison, the effects on GTPγS-untreated basal GIRK currents shown in Figure 2 are also shown. The magnitudes of inhibition of GIRK currents by sertraline were compared with the 3 mM Ba^2+^-sensitive current components. Each point and error bar represent the mean ± SEM of the percentage responses (*n* = 5 for GTPγS-injected oocytes and *n* = 16 for GTPγS-untreated oocytes). Current responses were measured at a membrane potential of −70 mV in an hK solution that contained 96 mM K^+^.

GIRK channels are activated by various G_i/o_-protein-coupled receptors through the direct action of G-protein βγ subunits released from the heterotrimeric G-protein complex [32], [33]. The effects of sertraline on GIRK channels activated by G-protein-coupled signaling mechanisms were further examined using GTPγS, a nonhydrolyzable GTP analog that maintains G-proteins in an activated state. Injection of GTPγS into *Xenopus* oocytes injected with GIRK1 and GIRK2 mRNAs increased inward currents with time and reached a steady-state level (516.0±123.7 nA, *n* = 5) as reported previously [49], [51]. The increased inward currents were completely blocked by 3 mM Ba^2+^, whereas GTPγS injection into uninjected oocytes had no significant effect on current responses to 3 mM Ba^2+^ (3.9±2.1 nA, *n* = 5). Increased GIRK currents composed of basal GIRK currents and GTPγS-induced GIRK currents were inhibited by sertraline (IC_25_ = 5.5±0.7 µM; IC_50_ = 18.1±3.0 µM; *n*_H_ = 1.24±0.09; *n* = 5; Fig. 5). The concentration response curve for the inhibition of total GIRK currents by sertraline was partially different from that for the inhibition of basal GIRK currents in GTPγS-untreated oocytes injected with GIRK1 and GIRK2 mRNAs (*P*<0.05 at 30 µM, Tukey-Kramer *post hoc* test, Fig. 5). The results suggest that the potency of the inhibition of GIRK channels activated by GTPγS-induced G-protein activation may be slightly higher than that of basally active GIRK channels, although the maximal efficacy was similar.

#### Sertraline inhibits ethanol-induced GIRK currents

**Figure 6:**
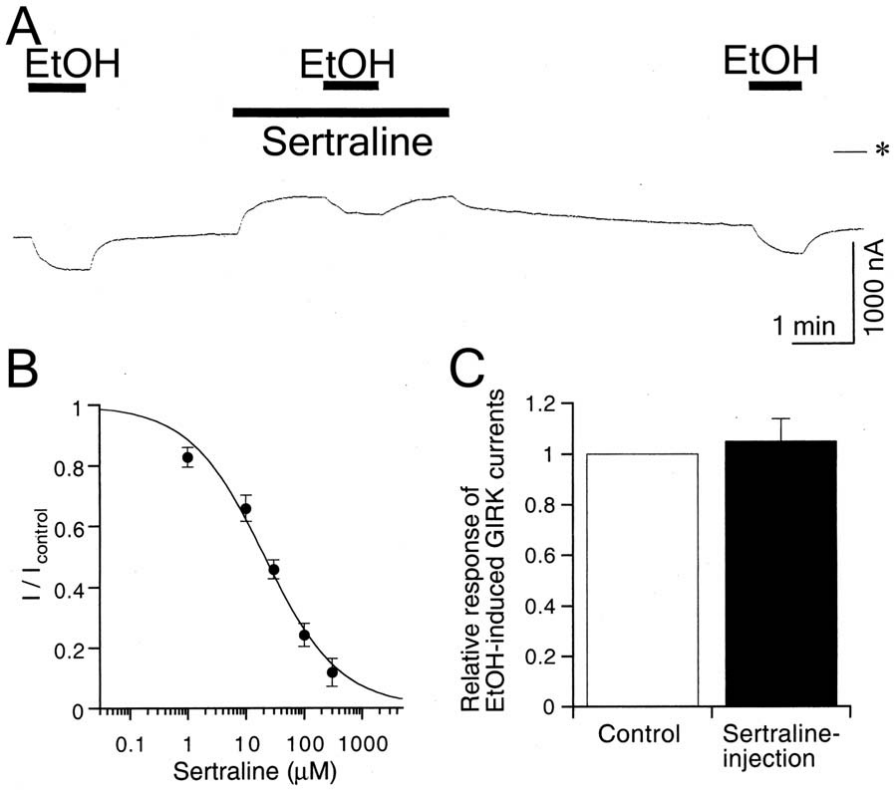
Effect of sertraline on ethanol-induced GIRK currents. (A) Current responses to 100 mM ethanol (EtOH), 100 mM EtOH in the presence of 30 µM sertraline, and 100 mM EtOH in an oocyte injected with GIRK1 and GIRK2 mRNAs. Asterisk indicates the zero current level. Bars show the duration of application. (B) Concentration-dependent inhibition of EtOH-induced GIRK currents by sertraline. *I*_control_ is the amplitude of GIRK currents induced by 100 mM EtOH (344.2±40.3 nA, *n* = 6), and *I* is the current amplitude in the presence of sertraline. (C) Lack of effect of intracellular sertraline on 100 mM EtOH-induced GIRK currents. The amplitude of EtOH-induced GIRK currents after sertraline injection (black bar) was compared with EtOH-induced GIRK currents before the injection (control, white bar) in the same oocyte that expressed GIRK channels (*n* = 5). Current responses were measured at a membrane potential of −70 mV in an hK solution that contained 96 mM K^+^. All values are expressed as mean ± SEM.

GIRK channels are also activated by ethanol independent of G-protein signaling pathways [34]. Sertraline was shown to reduce ethanol consumption in mice [52] and was effective in alcoholics [53]. Therefore, we also examined the effects of sertraline on GIRK channel activation induced by ethanol. The effects of sertraline were evaluated by measuring the amplitude of the ethanol-induced current response during the extracellular application of sertraline at different concentrations. In oocytes injected with GIRK1 and GIRK2 mRNAs, the GIRK currents induced by 100 mM ethanol (344.2±40.3 nA, *n* = 6) were reversibly attenuated in the presence of sertraline (IC_25_ = 6.2±1.4 µM; IC_50_ = 29.6±5.5 µM; *n*_H_ = 0.87±0.17; *n* = 6; Fig. 6A, 6B). However, the 100 mM ethanol-induced GIRK currents were not significantly affected by intracellularly applied sertraline (104.9±9.1% of untreated control current, paired *t*-test, *P*>0.1, *n* = 6; Fig. 6C). Moreover, in oocytes that expressed GIRK channels, the basal currents were not substantially affected by intracellularly applied sertraline (92.5±1.6% of untreated control current, *n* = 6). The results indicate that intracellular sertraline could not inhibit GIRK channels. In contrast, GIRK channel inhibition induced by extracellularly applied sertraline, which is mainly protonated at pH 7.4, was reversible with washout (Figs. 1A and 6A). Because the protonated form may not readily permeate the cell membrane, extracellularly applied sertraline may exist mainly on the extracellular side. Altogether, extracellular sertraline may inhibit GIRK channels activated by ethanol. Additionally, the extent of inhibition by sertraline of GIRK1/2 channels activated by ethanol was higher at 100 and 300 µM than that of basally active GIRK1/2 channels by G-proteins (*P*<0.05, Tukey-Kramer *post hoc* test), indicating a significant difference in the maximal efficacy of sertraline between ethanol activation of GIRK channels and G-protein activation of the channels.

## Discussion

The present study demonstrated that the SSRI sertraline, SNRI duloxetine, and second-generation TCA amoxapine effectively inhibited brain-type GIRK1/2 channels and cardiac-type GIRK1/4 channels expressed in *Xenopus* oocytes. However, the 5-HT_2_ receptor antagonist nefazodone, SNRI venlafaxine, and NaSSAs mianserin and mirtazapine weakly inhibited both types of GIRK channels even at high concentrations. The inhibitions by different classes of antidepressants were concentration-dependent with various degrees of potency and effectiveness. In contrast, Kir2.1 channels in other Kir channel subfamilies were insensitive to all of the drugs. Furthermore, the present results suggest that sertraline and duloxetine primarily inhibited GIRK channels at the holding potential of 0 mV before the voltage pulses. The effects of sertraline on GIRK channels were voltage-independent and time-independent during each voltage pulse, similar to the effects of various TCAs [42]. The effects of duloxetine decreased voltage-dependently with negative membrane potentials and time-dependently up to a steady current level in each voltage pulse, and the voltage-dependency was associated with a time-dependent decrease in the inhibition by duloxetine at more negative membrane potentials. The present results also suggest that the site of action on the channels may be extracellular. In contrast, blockade of GIRK channels by extracellular Ba^2+^ and Cs^+^, which occlude the pore of the open channel, increases concentration-dependently, voltage-dependently with negative membrane potentials, and time-dependently with a comparatively small effect on the instantaneous current but marked inhibition on the steady-state current at the end of the voltage pulses [27]. These observations suggest that sertraline and duloxetine may cause an allosteric conformational change in GIRK channels, rather than simple occlusion of the open channel. Additionally, sertraline may stably bind to the channels during the voltage pulses, whereas duloxetine may partially dissociate from the channels in the voltage pulses. The *n*_H_ values obtained from the concentration-response relationships for sertraline and duloxetine were almost 1 (Table 1), suggesting an one-to-one interaction between the drug and the binding site. Interestingly, GIRK channels were significantly inhibited by the SSRI sertraline and SNRI duloxetine, despite a great difference in the pharmacological profiles for monoamine transporters. The chemical structure of sertraline is distinct from that of duloxetine [2], [54]. These antidepressants may act at different binding sites on the channels, and agents with similar structures may interact with GIRK channels. However, the SNRIs venlafaxine and milnacipran [43] had weak or little effects on GIRK channels, respectively. The distinctive effects of the SNRIs on GIRK channels may be attribute to their diverse chemical structures [54]. The *Xenopus* oocyte expression system is useful to determine drug actions on membrane proteins, such as voltage-gated Na^+^ and Ca^2+^ channels, glutamate receptor channels, 5HT_1C_ receptor [55]. Since neuronal and cardiac GIRK channels are considered to consist predominantly of GIRK1/2 channels and GIRK1/4 channels, respectively [26], [29], [36], the effects of antidepressants on GIRK1/2 and GIRK1/4 channels expressed in *Xenopus* oocytes were investigated in the present study. However, GIRK subunits have been suggested to form functional GIRK channels composed of several types of tetrameric stoichimetries in various cell populations, particularly neurons [56]. GIRK1 subunits are posttranslationally modified by glycosylation [26], [56], [57]. Furthermore, GIRK channels are regulated by not only G proteins but also phosphatidylinositol 4,5-bisphosphate in the cell membrane, polyamines and protein kinases [33]. The effects of antidepressants on GIRK channels might be influenced by differences in composition of the channel subunits, levels of glycosylation of GIRK1 subunits, and interaction with membrane and intracellular factors between the *Xennopus* oocyte expression system and neurons. Further studies using neurons and cardiac myocytes may be useful for advancing our understanding of the effects of antidepressants on GIRK channels.

The therapeutic serum concentrations range from approximately 0.16 to 0.82 µM for sertraline, 0.07 to 0.27 µM for duloxetine, 0.57 to 1.9 µM for amoxapine, 0.02 to 0.64 µM for nefazodone, 0.06 to 0.26 µM for mianserin, 0.08 to 0.37 µM for mirtazapine, and 0.72 to 1.44 µM for venlafaxine [2], [58]–[60]. Additionally, increases in antidepressant doses are associated with increases in blood concentrations [59]. The concentrations in cases of overdose were reported to reach up to 13.7 µM for sertraline [61], 8.4 µM for duloxetine [62], 57.4 µM for amoxapine [63], 11.7 µM for nefazodone, 18.9 µM for mianserin [59], 8.7 µM for mirtazapine [64], and 302.8 µM for venlafaxine [65]. Most of the doses of antidepressants are distributed in various tissues from the blood, and antidepressants generally accumulate in the brain [2], [58], [66]. Indeed, brain levels of antidepressants were 40-fold higher for sertraline [67], 15-fold higher for duloxetine [68], 8.7- to 35.5-fold higher for amoxapine [69], 1.3- to 1.8-fold higher for nefazodone [70], 12.1-fold higher for mianserin [71], 3.2-fold higher for mirtazapine, and 4.9-fold higher for venlafaxine [66] compared with blood levels. Altogether, due to the high brain-to-blood partition ratios, presumed brain concentrations during treatment with therapeutic doses would range from approximately 6.4 to 32.8 µM for sertraline and 5.0 to 67.5 µM for amoxapine, and those after overdose would reach up to 548 µM for sertraline, 126 µM for duloxetine, 499 or 2038 µM for amoxapine, 229 µM for mianserin, and 1484 µM for venlafaxine. In addition, it has been shown that the therapeutic concentrations of some SSRIs in the brain were much higher than binding affinities of the antidepressants to monoamine transportors [72]–[75]. Brain concentrations at therapeutic doses of sertraline and amoxapine and after overdose of sertraline, duloxetine, amoxapine, mianserin and venlafaxine overlap with their effective concentrations in inhibiting predominant brain-type GIRK1/2 channels (Fig. 2). Therefore, the present results suggest that some inhibition of GIRK channels in the brain might occur with the antidepressant medication, particularly sertraline. However, mirtazapine and nefazodone may have small or little effects on GIRK channels even at toxic levels. Inhibition of GIRK channels causes a depolarization of membrane potential, resulting in an increase in cell excitability [38]. GIRK channels play an important role in regulating neuronal excitability and synaptic transmission [36], [41]. Therefore, even partial inhibition of GIRK channels by the antidepressants may affect various brain functions.

Interestingly, GIRK2 knockout mice exhibit reduced anxiety-related behavior [76]. Animal studies have shown that sertraline has anxiolytic properties [77], [78]. Indeed sertraline is clinically effective in the treatment of panic disorder and posttraumatic stress disorder [79]. Although the therapeutic effects are generally thought to be primarily attributable to inhibition of serotonin reuptake in the brain [2], some inhibition of GIRK channels might also contribute to improvement of anxiety symptoms.

Although the risk of seizures with antidepressants is generally very low, the association with overdose is well established [80]. However, the molecular mechanisms by which antidepressants cause seizures have not been clarified. GIRK2 knockout mice exhibit spontaneous seizures and are more susceptible to seizures induced by pentylenetetrazol than wild-type mice [37]. The risk of seizures in overdoses with sertraline, duloxetine, mianserin, and venlafaxine significantly increases [80]–[82], and amoxapine overdose is more likely to cause seizures [83]. Brain levels of the drugs in overdose cases may be considerably higher than levels during treatment at therapeutic doses, suggesting significant inhibition of neuronal GIRK channels by the drugs. Additionally, other types of K^+^ channels are inhibited by antidepressants at micromolar concentrations, that is, the two-pore-domain K^+^ channel, TREK-1 for sertraline and voltage-gated K^+^ channels for amoxapine and mianserin [16], [17], [21]. Therefore, the inhibition of GIRK channels by the drugs after overdose together with the different types of K^+^ channels may contribute to increased seizure activity and the occurrence of other neurological side effects by increasing neuronal excitability.

In the heart, GIRK channels cause a slowing of heart rate in response to activation of M_2_ muscarinic receptors through acetylcholine release from the stimulated vagus nerve [25], [26]. GIRK1 and GIRK4 knockout mice exhibit slightly elevated resting heart rates [39]. The present results indicate that sertraline, duloxetine, amoxapine, and venlafaxine can partially inhibit cardiac-type GIRK1/4 channels at blood levels after overdose, although the corresponding heart concentrations were not determined. These antidepressants are associated with sinus tachycardia in cases of toxicity after overdose [81], [82], [84], [85]. In addition, the drugs exhibit low micromolar binding affinities for the muscarinic receptor, with the exception of venlafaxine [2], [86], and nanomolar to low micromolar binding affinities for norepinephrine transporters [2], [68]. Altogether, sinus tachycardia associated with drug overdose may be related to partial inhibition of atrial GIRK channels as well as antagonism of the muscarinic receptor and enhancement of sympathetic nerve activity.

Sertraline was shown to be effective in the treatment of alcoholics [53]. Interestingly, GIRK2 knockout mice show reduced ethanol-induced conditioned taste aversion and conditioned place preference and are less sensitive than wild-types to some of the acute effects of ethanol, including anxiolysis, habituated locomotor stimulation, and acute handling-induced convulsions [76], [87]. In the present study, sertraline inhibited ethanol-induced GIRK1/2 currents. Sertraline may suppress some of the GIRK-related effects of ethanol. Furthermore, GIRK knockout mice show an attenuation of the morphine withdrawal syndrome [88]. Sertraline reduced the severity of the naloxone-precipitated opioid withdrawal syndrome in rats [89]. GIRK knockout mice also show reduced cocaine self-administration [90]. Inhibition of GIRK channels by sertraline may play a role in the treatment of addiction to these drugs.

## Supporting Information

Figure S1 **Effect of sertraline on outward GIRK currents. In a** ***Xenopus*** **oocyte injected with GIRK1 and GIRK2 mRNAs, current responses to 30 µM sertraline and 3 mM Ba^2+^ at a membrane potential of −10 mV in a K4 solution that contained 4 mM K^+^ are shown.** Asterisk indicates the zero current level.
(DOC)

## References

[1] Kent JM (2000). SNaRIs, NaSSAs, and NaRIs: new agents for the treatment of depression.. Lancet.

[2] Baldessarini RJ, Hardman JG, Limbird LE, Gilman AG (2001). Drugs and the treatment of psychiatric disorders: depression and anxiety disorders.. Goodman & Gilman's The Pharmacological Basis of Therapeutics. 10th edn.

[3] Mann JJ (2005). The management of depression.. N Engl J Med.

[4] Ni YG, Miledi R (1997). Blockage of 5HT_2C_ serotonin receptors by fluoxetine (Prozac).. Proc Natl Acad Sci USA.

[5] Fan P (1994). Effects of antidepressants on the inward current mediated by 5-HT_3_ receptors in rat nodose ganglion neurones.. Br J Pharmacol.

[6] Shytle RD, Silver AA, Lukas RJ, Newman MB, Sheehan DV (2002). Nicotinic acetylcholine receptors as targets for antidepressants.. Mol Psychiatry.

[7] Sernagor E, Kuhn D, Vyklicky L, Mayer ML (1989). Open channel block of NMDA receptor responses evoked by tricyclic antidepressants.. Neuron.

[8] Nakazawa K, Inoue K, Ohno Y (1999). Block and unblock by imipramine of cloned and mutated P2X_2_ receptor/channel expressed in *Xenopus* oocytes.. Neurosci Lett.

[9] Ogata N, Yoshii M, Narahashi T (1989). Psychotropic drugs block voltage-gated ion channels in neuroblastoma cells.. Brain Res.

[10] Mathie A, Wooltorton JRA, Watkins CS (1998). Voltage-activated potassium channels in mammalian neurons and their block by novel pharmacological agents.. Gen Pharmacol.

[11] Pancrazio JJ, Kamatchi GL, Roscoe AK, Lynch C (1998). Inhibition of neuronal Na^+^ channels by antidepressant drugs.. J Pharmacol Exp Ther.

[12] Teschemacher AG, Seward EP, Hancox JC, Witchel HJ (1999). Inhibition of the current of heterologously expressed HERG potassium channels by imipramine and amitriptyline.. Br J Pharmacol.

[13] Deák F, Lasztóczi B, Pacher P, Petheö GL, Kecskeméti V (2000). Inhibition of voltage-gated calcium channels by fluoxetine in rat hippocampal pyramidal cells.. Neuropharmacology.

[14] Choi BH, Choi J-S, Yoon SH, Rhie D-J, Min DS (2001). Effects of norfluoxetine, the major metabolite of fluoxetine, on the cloned neuronal potassium channel Kv3.1.. Neuropharmacology.

[15] Cuellar-Quintero JL, García DE, Cruzblanca H (2001). The antidepressant imipramine inhibits the M-type K^+^ current in rat sympathetic neurons.. Neuroreport.

[16] Scherer D, von Löwenstern K, Zitron E, Scholz EP, Bloehs R (2008). Inhibition of cardiac hERG potassium channels by tetracyclic antidepressant mianserin.. Naunyn-Schmiedeberg's Arch Pharmacol.

[17] He Y-L, Zhan X-Q, Yang G, Sun J, Mei Y-A (2010). Amoxapine inhibits the delayed rectifier outward K^+^ current in mousse cortical neurons via cAMP/protein kinase A pathways.. J Pharmacol Exp Ther.

[18] Lee K, McKenna F, Rowe ICM, Ashford MLJ (1997). The effects of neuroleptic and tricyclic compounds on BK_Ca_ channels activity in rat isolated cortical neurones.. Br J Pharmacol.

[19] Dreixler JC, Bian J-T, Cao Y-J, Roberts MT, Roizen JD (2000). Block of rat brain recombinant SK channels by tricyclic antidepressants and related compounds.. Eur J Pharmacol.

[20] Terstappen GC, Pula G, Carignani C, Chen MX, Roncarati R (2001). Pharmacological characterisation of the human small conductance calcium-activated potassium channel hSK3 reveals sensitivity to tricyclic antidepressants and antipsychotic phenothiazines.. Neuropharmacology.

[21] Heurteaux C, Lucas G, Guy N, Yacoubi ME, Thümmler S (2006). Deletion of the background potassium channel TREK-1 results in a depression-resistant phenotype.. Nat Neurosci.

[22] Maertens C, Droogmans G, Verbesselt R, Nilius B (2002). Block of volume-regulated anion channels by selective serotonin reuptake inhibitors.. Naunyn-Schmiedeberg's Arch Pharmacol.

[23] Maertens C, Wei L, Voets T, Droogmans G, Nilius B (1999). Block by fluoxetine of volume-regulated anion channels.. Br J Pharmacol.

[24] Reimann F, Ashcroft FM (1999). Inwardly rectifying potassium channels.. Curr Opin Cell Biol.

[25] Kubo Y, Reuveny E, Slesinger PA, Jan YN, Jan LY (1993). Primary structure and functional expression of a rat G-protein-coupled muscarinic potassium channel.. Nature.

[26] Krapivinsky G, Gordon EA, Wickman K, Velimirovic B, Krapivinsky L (1995). The G-protein-gated atrial K^+^ channel I_KACh_ is a heteromultimer of two inwardly rectifying K^+^-channel proteins.. Nature.

[27] Lesage F, Guillemare E, Fink M, Duprat F, Heurteaux C (1995). Molecular properties of neuronal G-protein-activated inwardly rectifying K^+^ channels.. J Biol Chem.

[28] Karschin C, Dißmann E, Stuhmer W, Karschin A (1996). IRK(1–3) and GIRK(1–4) inwardly rectifying K^+^ channel mRNAs are differentially expressed in the adult rat brain.. J Neurosci.

[29] Liao YJ, Jan YN, Jan LY (1996). Heteromultimerization of G-protein-gated inwardly rectifying K^+^ channel proteins GIRK1 and GIRK2 and their altered expression in *weaver* brain.. J Neurosci.

[30] Inanobe A, Yoshimoto Y, Horio Y, Morishige K-I, Hibino H (1999). Characterization of G-protein-gated K^+^ channels composed of Kir3.2 subunits in dopaminergic neurons of the substantia nigra.. J Neurosci.

[31] North RA (1989). Drug receptors and the inhibition of nerve cells.. Br J Pharmacol.

[32] Dascal N (1997). Signalling via the G protein-activated K^+^ channels.. Cell Signal.

[33] Kobayashi T, Ikeda K (2006). G protein-activated inwardly rectifying potassium channels as potential therapeutic targets.. Curr Pharm Des.

[34] Kobayashi T, Ikeda K, Kojima H, Niki H, Yano R (1999). Ethanol opens G-protein-activated inwardly rectifying K^+^ channels.. Nat Neurosci.

[35] Lewohl JM, Wilson WR, Mayfield RD, Brozowski SJ, Morrisett RA (1999). G-protein-coupled inwardly rectifying potassium channels are targets of alcohol action.. Nat Neurosci.

[36] Lüscher C, Jan LY, Stoffel M, Malenka RC, Nicoll RA (1997). G protein-coupled inwardly rectifying K^+^ channels (GIRKs) mediate postsynaptic but not presynaptic transmitter actions in hippocampal neurons.. Neuron.

[37] Signorini S, Liao YJ, Duncan SA, Jan LY, Stoffel M (1997). Normal cerebellar development but susceptibility to seizures in mice lacking G protein-coupled, inwardly rectifying K^+^ channel GIRK2.. Proc Natl Acad Sci USA.

[38] Kuzhikandathil EV, Oxford GS (2002). Classic D1 dopamine receptor antagonist *R*-(+)-7-chloro-8-hydroxy-3-methyl-1-phenyl-2,3,4,5-tetrahydro-1*H*-3-benzazepine hydrochloride (SCH23390) directly inhibits G protein-coupled inwardly rectifying potassium channels.. Mol Pharmacol.

[39] Bettahi I, Marker CL, Roman MI, Wickman K (2002). Contribution of the Kir3.1 subunit to the muscarinic-gated atrial potassium channel I_KACh_.. J Biol Chem.

[40] Hashimoto N, Yamashita T, Tsuruzoe N (2006). Tertiapin, a selective IK_ACh_ blocker, terminates atrial fibrillation with selective atrial effective refractory period prolongation.. Pharmacol Res.

[41] Lüscher C, Slesinger PA (2010). Emerging roles for G protein-gated inwardly rectifying potassium (GIRK) channels in health and disease.. Nat Rev Neurosci.

[42] Kobayashi T, Washiyama K, Ikeda K (2004). Inhibition of G protein-activated inwardly rectifying K^+^ channels by various antidepressant drugs.. Neuropsychopharmacology.

[43] Kobayashi T, Washiyama K, Ikeda K (2006). Inhibition of G protein-activated inwardly rectifying K^+^ channels by the antidepressant paroxetine.. J Pharmacol Sci.

[44] Kobayashi T, Ikeda K, Ichikawa T, Abe S, Togashi S (1995). Molecular cloning of a mouse G-protein-activated K^+^ channel (mGIRK1) and distinct distributions of three GIRK (GIRK1, 2 and 3) mRNAs in mouse brain.. Biochem Biophys Res Commun.

[45] Kobayashi T, Ikeda K, Kumanishi T (2000). Inhibition by various antipsychotic drugs of the G-protein-activated inwardly rectifying K^+^ (GIRK) channels expressed in *Xenopus* oocytes.. Br J Pharmacol.

[46] Kubo Y, Baldwin TJ, Jan YN, Jan LY (1993). Primary structure and functional expression of a mouse inward rectifier potassium channel.. Nature.

[47] Kobayashi T, Ikeda K, Kumanishi T (2002). Functional characterization of an endogenous *Xenopus* oocyte adenosine receptor.. Br J Pharmacol.

[48] Ikeda K, Yoshii M, Sora I, Kobayashi T (2003). Opioid receptor coupling to GIRK channels: in vitro studies using a Xenopus oocyte expression system and in vivo studies on weaver mutant mice.. Methods Mol Med.

[49] Kovoor A, Henry DJ, Chavkin C (1995). Agonist-induced desensitization of the mu opioid receptor-coupled potassium channel (GIRK1).. J Biol Chem.

[50] Kobayashi T, Washiyama K, Ikeda K (2003). Inhibition of G protein-activated inwardly rectifying K^+^ channels by fluoxetine (Prozac).. Br J Pharmacol.

[51] Kobayashi T, Washiyama K, Ikeda K (2010). Inhibition of G-protein-activated inwardly rectifying K^+^ channels by the selective norepinephrine reuptake inhibitors atomoxetine and reboxetine.. Neuropsychopharmacology.

[52] Gulley JM, McNamara C, Barbera TJ, Ritz MC, George FR (1995). Selective serotonin reuptake inhibitors: effects of chronic treatment on ethanol-reinforced behavior in mice.. Alcohol.

[53] Pettinati HM, Volpicelli JR, Kranzler HR, Luck G, Rukstalis MR (2000). Sertraline treatment for alcohol dependence: interactive effects of medication and alcoholic subtype.. Alcohol Clin Exp Res.

[54] Stahl SM, Grady MM, Moret C, Briley M (2005). SNRIs: their pharmacology, clinical efficacy, and tolerability in comparison with other classes of antidepressants.. CNS Spectr.

[55] Sigel E (1990). Use of *Xenopus* oocytes for the functional expression of plasma membrane proteins.. J Membrane Biol.

[56] Aguado C, Colón J, Ciruela F, Schlaudraff F, Cabanero MJ (2008). Cell type-specific subunit composition of G protein-gated potassium channels in the cerebellum.. J Neurochem.

[57] Pabon A, Chan KW, Sui JL, Wu X, Logothetis DE (2000). Glycosylation of GIRK1 at Asn^119^ and ROMK1 at Asn^117^ has different consequences in potassium channel function.. J Biol Chem.

[58] Musshoff F, Padosch S, Steinborn S, Maeda SSB (2004). Fatal blood and tissue concentrations of more than 200 drugs.. Forensic Sci Int.

[59] Welzen M, Uges DRA (2004). TIAFT reference blood level list of the therapeutic and toxic substances.. http://www.tiaft.org.

[60] Lobo ED, Bergstrom RF, Reddy S, Quinlan T (2008). In vitro and in vivo evaluations of cytochrome P450 1A2 interactions with duloxetine.. Clin Pharmacokinet.

[61] Goeringer KE, Raymon L, Christian GD, Logan BK (2000). Postmortem forensic toxicology of selective serotonin reuptake inhibitors: a review of pharmacology and report of 168 cases.. J Forensic Sci.

[62] Vey EL, Kovelman I (2010). Adverse events, toxicity and post-mortem data on duloxetine: case reports and literature survey.. J Forensic Legal Med.

[63] Taylor RL, Crooks CR, Caplan YH (1982). The determination of amoxapine in human fatal overdoses.. J Anal Toxicol.

[64] Holzbach R, Jahn H, Pajonk FG, Mähne C (1998). Suicide attempts with mirtazapine overdose without complications.. Biol Psychiatry.

[65] Levine B, Jenkins AJ, Queen M, Jufer R, Sialek JE (1996). Distribution of venlafaxine in three postmortem cases.. J Anal Toxicol.

[66] Uhr M, Grauer MT, Holsboer F (2003). Differential enhancement of antidepressant penetration into the brain in mice with abcb1ab (mdr1ab) P-glycoprotein gene distruption.. Biol Psychiatry.

[67] Tremaine LM, Welch WM, Ronfeld RA (1989). Metabolism and disposition of the 5-hydroxytriptamine uptake blocker sertraline in the rat and dog.. Drug Metab Dispos.

[68] Bymaster FP, Lee TC, Knadler MP, Detke MJ, Iyengar S (2005). The dual transporter inhibitor duloxetine: a review of its preclinical pharmacology, pharmacokinetic profile, and clinical results in depression.. Curr Pharm Des.

[69] Sedgwick P, Spiehler VR, Lowe DR (1982). Toxicological findings in amoxapine overdose.. J Anal Toxicol.

[70] Nacca A, Guiso G, Fracasso C, Cervo L, Caccia S (1996). Brain-to-blood partition and *in vivo* inhibition 5-hydroxytryptamine reuptake and quipazine-mediated behaviour of nefazodone and its main active metabolites in rodents.. Br J Pharmacol.

[71] Altamura AC, De Novelis F, Mauri MC, Gomeni R (1987). Plasma and brain pharmacokinetics of mianserin after single and multiple dosing in mice.. Prog Neuropsychopharmacol Biol Psychiatry.

[72] Karson CN, Newton JEO, Livingston R, Jolly JB, Cooper TB (1993). Human brain fluoxetine concentrations.. J Neuropsychiatry Clin Neurosci.

[73] Bolo NR, Hode Y, Nedelec J-F, Laine E, Wagner G (2000). Brain pharmacokinetics and tissue distribution *in vivo* of fluvoxamine and fluoxetine by fluorine magnetic resonance spectroscopy.. Neuropsychopharmacology.

[74] Henry ME, Moore CM, Kaufman MJ, Michelson D, Schmidt ME (2000). Brain kinetics of paroxetine and fluoxetine on the third day of placebo substitution: a fluorine MRS study.. Am J Psychiatry.

[75] Henry ME, Schmidt ME, Hennen J, Villafuerte RA, Butman ML (2005). A comparison of brain and serum pharmacokinetics of *R*-fluoxetine and racemic fluoxetine: a 19-F MRS study.. Neuropsychopharmacology.

[76] Blednov YA, Stoffel M, Chang SR, Harris RA (2001). Potassium channels as targets for ethanol: studies of G-protein-coupled inwardly rectifying potassium channel 2 (GIRK2) null mutant mice.. J Pharmacol Exp Ther.

[77] Sánchez C, Meier E (1997). Behavioral profiles of SSRIs in animal models of depression, anxiety and aggression.. Psychopharrmacology.

[78] Harada Y, Kohara N, Imaeda T (2006). Pharmacological, pharmacokinetic, and clinical profile of sertraline hydrochloride (J ZOLOFT™).. Folia Pharmacol Jpn.

[79] Schatzberg AF (2000). New indications for antidepressants.. J Clin Psychiatry.

[80] Montgomery SA (2005). Antidepressants and seizures: emphasis on newer agents and clinical implications.. Int J Clin Pract.

[81] Whyte IM, Dawson AH, Buckley NA (2003). Relative toxicity of venlafaxine and selective serotonin reuptake inhibitors in overdose compared to tricyclic antidepressants.. Q J Med.

[82] Isbister GK, Bowe SJ, Dawson A, Whyte IM (2004). Relative toxicity of selective serotonin reuptake inhibitors (SSRIs) in overdose.. J Toxicol Clin Toxicol.

[83] Litovitz TL, Troutman WG (1983). Amoxapine overdose: seizures and fatalities.. JAMA.

[84] Frommer DA, Kulig KW, Marx JA, Rumack B (1987). Tricyclic antidepressant overdose: a review.. JAMA.

[85] Lott RS, Baker DE (2003). Duloxetine: a new antidepressant.. Advances Pharmacy.

[86] Bymaster FP, Dreshfield-Ahmad LJ, Threlkeld PG, Shaw JL, Thompson L (2001). Comparative affinity of duloxetine and venlafaxine for serotonin and norepinephrine transporters in vitro and in vivo, human serotonin receptor subtypes, and other neuronal receptors.. Neuropsychopharmacology.

[87] Hill KG, Alva H, Blednov YA, Cunningham CL (2003). Reduced ethanol-induced conditioned taste aversion and conditioned place preference in GIRK2 null mutant mice.. Psychopharmacology.

[88] Cruz HG, Berton F, Sollini M, Blanchet C, Pravetoni M (2008). Absence and rescue of morphine withdrawal in KIR/Kir3 knock-out mice.. J Neurosci.

[89] Gray AM (2002). The effect of fluvoxamine and sertraline on the opioid withdrawal syndrome: a combined in vivo cerebral microdialysis and behavioural study.. Eur Neuropschopharmacol.

[90] Morgan AD, Carroll ME, Loth AK, Stoffel M, Wickman K (2003). Decreased cocaine self-administration in Kir3 potassium channel subunit knockout mice.. Neuropsychopharmacology.

