# Supplementary material for: Inhibition of G Protein-Activated Inwardly Rectifying K+ Channels by Different Classes of Antidepressants
Source: PLoS One. 2011 Dec 2;6(12):e28208. doi: 10.1371/journal.pone.0028208 (PMC3229538; doi:10.1371/journal.pone.0028208)
Supplement: Figure S1 — Effect of sertraline on outward GIRK currents. In a Xenopus oocyte injected with GIRK1 and GIRK2 mRNAs, current responses to 30 µM sertraline and 3 mM Ba2+ at a membrane potential of −10 mV in a K4 solution that contained 4 mM K+ are shown. Asterisk indicates the zero current level. (DOC) [file pone.0028208.s001.doc]

# **Supplementary Information**


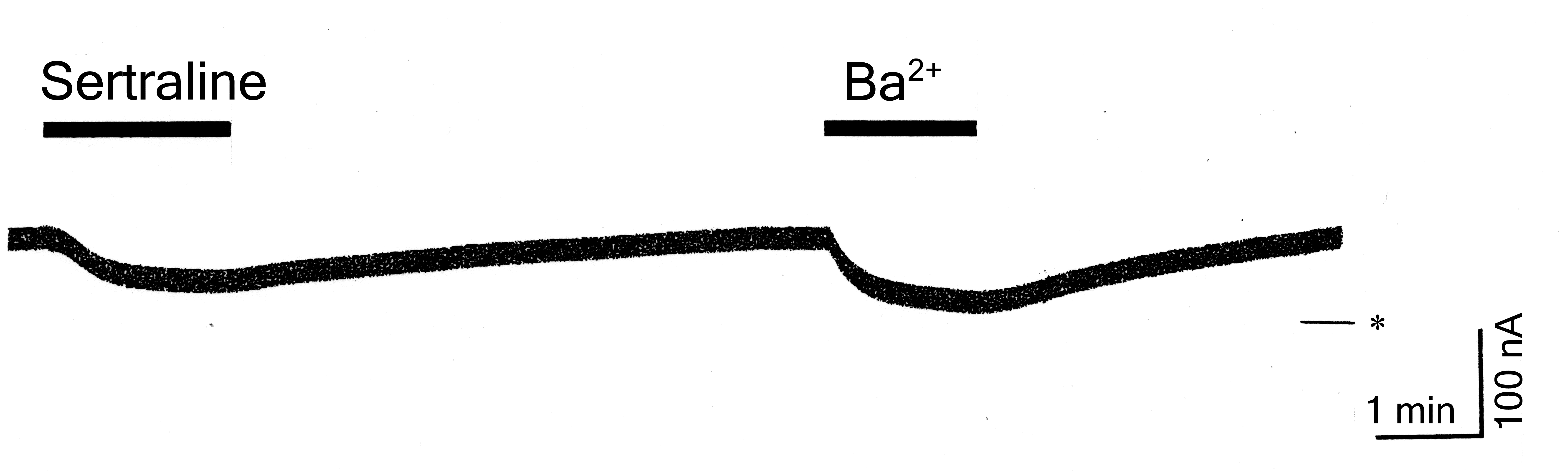


**Figure S1** Effect of sertraline on outward GIRK currents. In a *Xenopus* oocyte injected with GIRK1 and GIRK2 mRNAs, current responses to 30 μM sertraline and 3 mM Ba^2+^ at a membrane potential of −10 mV in a K4 solution that contained 4 mM K^+^ are shown. Asterisk indicates the zero current level.
